# A Review of Dental Histology

**Published:** 1858-04

**Authors:** J. Smith Dodge


### ARTICLE II.

*A Review of Dental Histology.* By J. SMITH DODGE, JR., M. D., D. D. S.

THE teeth have many claims to more careful notice than physiologists have been accustomed to give them. Much might be said of the great variety of uses which they are made to subserve in the different orders of animals; and of the minute though important differences which render them, in each species, so perfect an index of the peculiar habits of the animal. It is extremely interesting, also, to observe how cunningly the proportions and the positions of the component tissues, are varied to answer the destined end, and to render for many years inoperative, the tendency which such organs necessarily have, to lose by attrition their original and typical form. The relative position, the shape, and the replacement, of the teeth might all be profitably dwelt on. But I propose to confine myself to the human teeth; and in them to consider some points in the histology and internal anatomy of the organs. If anything of novelty can be claimed for this paper, it must be found rather in the mode of treating the subject, than in any peculiar views advanced.

The teeth offer many points of striking contrast with other parts of the body: the requirements to be answered in these organs are so unique, and yet so seemingly analogous to what is elsewhere demanded, that nature appears, to the superficial observer, to have contradicted herself—a thing which a true philosophy can never admit. For instance, the masticating functions of the teeth implies repeated and severe friction; now many parts of the system are laid under the same necessity—as, the articulations, the palmar and plantar surfaces—and in them the unvarying plan is, to provide a surface capable of bearing temporary friction, but easily yielding to it, and as readily renewed; nature triumphs by persistency rather than by resistance. But in the case of the teeth, not only is a denser surface actually required for the due performance of function, but, as we shall presently see, the more ordinary plan would offer no resistance to the action of buccal acids ; and again, continual renewal of surface requires a high degree of vitality in the tissues immediately concerned in its production; and this, acted on by the frequent mastications which nutrition demands, would strongly predispose to morbid irritability and inflammation, in organs whose integrity and power of action is of the highest moment to the whole organism. Here then, nature, bound by no shackles but the end to be attained, utterly deserting the Fabian policy, strikes boldly into the only other course; and a substance is produced so hard as to take the highest polish, and to resist the wear of daily mastication for very many years.

Another cause, however, would compel to the same result, for the teeth are destined to be always in a bath capable of acting chemically on their substance. Digestion requires that the parotidean and submaxillary fluid, destined to inaugurate, by its action on starch, the long series of assimilative processes, should be alkaline; and as constant immersion in such a menstruum, would in the end render soluble the albuminoid element of the teeth, neutralising acid is poured from the minute crypts of the mucous membrane. But such nicely adjusted balances are always easily disturbed, and accordingly, the varying conditions of the system cause alternately the one principle or the other to predominate. Happily, however, our frames are not, like so much of human mechanism, adapted only for unvarying favorable circumstances; but in every organ, and every tissue, is provision made to resist the temporary derangements of the normal state. But how could organs of the consistence, say, of horn, or even of dense osseous tissue, resist these fluctuations from alkaline to acid, the one destroying the tone, and finally the vitality, of the animal basis, the other stealthily abstracting molecule after molecule of earthy material? It is true, the entire alimentary tract is subject to acid action, and the stomach resists successfully a much intenser acid than often attacks the teeth; but these surfaces are immediately vitalized and readily renewed, by a subjacent layer whose vascularity and innervation present almost the acme of intensity in organic life; and I have briefly shown the impracticability of such a constitution in masticating organs.

Again, then, must nature triumph over what seem to us difficulties, and again is the triumph wonderfully complete. No doubt the calcified "membrane of Nasmyth," contributes to this result, by opposing the fissure of the enamel on sudden changes of temperature; so too, the trace of fluorine, which resists the solvent power of carbonic acid, and the great density of the tissue itself, which hinders the percolation of dissolving agents: but I cannot doubt that the most efficient and constant protection is the beautiful polish which is natural to the surface of the teeth. Such a surface is well known to prevent the action of powerful menstrua upon much easier dissolved substances than enamel.

Still another obstacle remains to the perfect operation of these indispensable organs: great density is incompatible with a high degree of vitality, and what is not highly vitalised the economy is prone to throw off on slight provocation. For this reason the bones, although so thoroughly traversed by bloodvessels, yet are everywhere invested and protected by highly vital tissues, which in some mysterious way seem to lend to less favored structures both pabulum and vital force—a process seen also in many phases of cell-development, and particularly conspicuous in the nutrition of cartilage. But the teeth, of so dense structure, must stand in a great measure exposed, subject to repeated shocks and the insidious attacks of dissolving fluids, yet the perfect tooth may go down to the grave with its aged owner.

This unique achievement is attained by a gradation of tissues in the organ, so that while the enamel presents all the hardness required by its various offices, while the dentine which supports it has no mean degree of vitality, and the cementum offers that necessary grade of organization which the softer tissues in contact may without reluctance tolerate, there resides within the tooth a structure so delicate and sensitive, so replete with the principle of life, that not only does it radiate a strong preserving influence throughout the dentine, but it may, by its slow and protracted suffering, derange the very centres of organic life, or in its maddening distress convulse the utmost limits of the system. How different is this wonderful and delicate organ from the vulgar idea of a tooth—an inorganic peg, a splinter of bone, useful to chew upon and thrust into the jaw by *gomphosis!*

In considering more at length these several tissues, I shall begin with the dentine, as being the most essential part of the tooth.

Dentine constitutes the principal substance of each tooth, not only in point of bulk, but also in respect to function. The pulp may ossify, the enamel may become abraded, yet the tooth retains a large portion of its original efficiency: without its support the enamel crumbles readily, and no one covets an exposed dental pulp.

What is its nature? Many considerations authorize us to call dentine modified bone. True, its microscopic structure and chemical constitution differ much from those of the larger bony masses, and its development is peculiar; but I shall endeavor to show that none of these considerations can avail to exclude it from the class of bones. Space is not at my command to present in detail each of these points, but I shall so far refer to them as to illustrate my views of the histological and mutual relations of dentine and the other dental tissues.

Mr. Tomes, who regards the three calcified tissues of teeth as but modifications of the same structure, speaks at length of the close relation of dentine to bone; and his description of the dental pulp, immediately before the commencement of calcification, would of itself almost establish the identity of the two tissues. He says, p. 92, "I think I shall be able to show you that the pulp is a modification of cartilage, modified to suit its peculiar position and function, and that the development of dentine is in every way similar to the development of bone:'' and to any one accustomed to the microscopic appearance of temporary cartilage when about to ossify, the diagrams given by Mr. Tomes, would alone be strong evidence of this position. But careful as are the observations of both, Mr. Nasmyth's description of the pulp at this stage, seems strangely at variance with the one just quoted; although there seems to be a few common points, which possibly indicate the way to a reconciliation of the two views.

The teeth are sometimes said to be products of the mucous membrane, and therefore not comparable with bone; but this is not true. The tooth-germ is covered at first with mucous membrane, but it originates and develops beneath that covering, and receives from it only the enamel, as we shall presently see.

Looking next at the structure of completed dentine, the superficial appearance under the microscope is, to be sure, very different from that of common bone; but if this were the end of microscopic study, the instrument would well deserve its vulgar appellation of "philosophic toy.'' The dentine has normally no Haversian canals, neither have the trabeculæ of the cancellar form of bony tissue; it has no lacunæ, but these can only be regarded as expansions of the canaliculi, designed to serve as depots of nutrient matter, and are surely not essential to the bony structure; if a greater density is required, and the nourishment of the tissue is provided for without abstracting from its dense material space for the reservoirs, can such a modification destroy the identity of a tissue? As regards the difference of dentinal tubuli from the canaliculi of ordinary bone, I suppose no one would base an argument on this alone; for if we regard them as really tubes, they correspond to the usual view of the canaliculi; or if we receive the view of Nasmyth, and consider them as solid fibres, this might give additional support to the views of those physiologists who believe the bony canals to have a similar character.

The chemical composition of dentine differs from that of bone, merely in the proportion of ingredients; and we find a similar, though less, variation in the varieties of ordinary bone, the phosphate of lime being a little greater in those of harder texture; which fact is sufficient to refute any argument based on the analysis of dentine.

The frame of the tooth, then, is bone. But even this densest variety of bone would be unequal to the prolonged friction to which the organ is necessarily subject. Besides, although dentine has normally no interstitial vascularity, it is abundantly supplied with the other element of vital connection, nerves. Nasmyth calls this tissue "extravascular and extranervous;" but although I am not aware that nervous fibrils have been miscroscopically demonstrated in the dentine, yet no one of common dental observation can doubt its extreme capability of sensation: and how could such a tissue endure the constant presence of irritants in contact with it? Hence the necessity of a covering, an *enamel*, of different physical and vital properties, namely, greater hardness, with whatever other arrangement may enable it to endure much friction, and absolute insensibility: and such are the distinctive characters of enamel.

But what are the histological relations of this singular tissue? Tomes, as I have already stated, regards this, as well as the two other hard tissues of the teeth, as a modification of bone, but it seems to me plainly obvious that this is an incorrect view of the matter. To be sure, the qualitative analysis is almost the same, but that is explicable on the principle that certain elements have been allotted to the human constitution, and of these the only ones suitable for a dense structure, must necessarily enter into all tissues having that character; for nature delights in producing many results with few means. But it is mainly the mode of formation of enamel, which points out its histology: it is clearly modified epithelium of mucous membrane. And analogy is much in favor of this view, for almost all the other hard protuberances of the higher animals are of similar origin: indeed, the histological resemblance of the horns of domestic animals to teeth, is not slight, the bony core answering to dentine, the true horn to enamel. And this view removes the startling anomaly of the naked protrusion of a tissue usually carefully covered and hidden, as is the osseous. The design according to which enamel was constructed seems to have been that of a tissue so formed as to maintain its position and function by its mere physical qualities—as it were, a lifeless product of the living system, a machine cunningly built in the inner chambers of the frame, and when wrought and burnished, thrust forth like the tortoise of ancient warfare, to cover and shield the life beneath. And yet this covering is not wholly beyond the reach of vitality, for it certainly receives calcareous matter as life advances, but probably rather by a simple imbibition from the dentinal surface, than by any elaborate assimilation. Besides, there exist in the stratum of enamel immediately next the dentine, prolongations of the dentinal tubuli, expanded into bulbs, to which is no doubt due, as has been suggested by Prof. Johnston, the extreme pain often produced in excavating shallow cavities, in which these bulbs have become exposed, but are still alive.

It is not merely the hardness of the enamel which gives to it its extraordinary power of resistance, but also its texture. Every one knows that its structure is columnar—rudely imaged by that of basaltic rock—and that the ends of the columns are presented to the attrition of the other teeth; and whoever has made various sections of enamel, has learned how much more easily it grinds away in a plane corresponding with the direction of the fibres than in one crossing them. But it is not generally stated, though it is obvious enough under the microscope, that those fibres run, not like basaltic columns, but at various angles with each other, interlacing more or less frequently, and even sometimes seeming twisted together like the fibres in a twine; as is shown in the fossil enamel of the mastodon, and also in that of the dog and other carnivora; and even in man the interlacing is sometimes very considerable. By such an arrangement much mutual support must be afforded by the enamel fibres, and the resistance of the whole mass be much increased.

There remains another element of the tooth, of bony constitution—the cement. Nothing can be more striking than the immense contrast which this tissue presents in the teeth of various animals, as regards its proportion and apparent importance; for while in many species it pervades the entire tooth largely, and forms even the majority of their bulk, in our own dental organs it is reduced, on the crown, to that extremely delicate layer known as "Nasmyth's membrane," and on the fang to a mere coating, somewhat thickened towards its lower end. Yet even in this condition, so little imposing as to seem merely rudimentary, it manifestly fulfils such important functions as would have seemed to justify its special creation. The layer which invests the enamel was named by Nasmyth, who first pointed it out, the "persistent dental capsule," although he evidently believed it to be only a prolongation of the cement, and even informs us that he has observed in it, upon the tooth of the ox, the specific lacunæ of bone. But even though this observation were lacking, the investment of the crown so much resembles the thin upper portions of the acknowledged cementum, which is equally without lacunæ, that it seems entirely safe to call the membrane of Nasmyth a prolongation of the investment of the fang. The identity of these structures seems to be demonstrated by a tooth in the museum of the Baltimore Dental College, in which has occurred an hypertrophy not only of the cementum on the fang, but also of this same "persistent dental capsule;" so that the entire tooth is pervaded by this abnormal growth, which, as Prof. Harris says, Principles, &c., p. 123, "must have commenced simultaneously with the commencement of the deposition of earthy salts in the dentinal pulp; and so rapidly did it proceed, that it completely broke up the enamel organ, penetrating every part of it, so that only here and there, embedded in its substance, small particles of enamel are seen." Let me now apply to this tooth the following remarks from Tomes, p. 104: "The enamel pulp and the dentine pulp, attain the full size of the parts for the development of which they are destined, before calcification of their substance commences. Not so, however, with the cement pulp; this increases on the external surface as calcification is progressing from within outwards, the former action supplying and keeping slightly in advance of, the latter." Now suppose such an action as this last to commence prematurely on the surface of the enamel organ, and to progress with the astonishing vigor which disease sometimes manifests; obviously, the rapidly formed cement would thrust itself into the immature enamel, and surround these small portions already calcified, producing the condition described by Prof. Harris.

It may be interesting, in this place, to show that the same membrane from which the cement-pulp is secreted about the fang, exists also just without the enamel organ. And I trust I may be excused if I quote rather at length from that excellent dental anatomist, Delabarre. He says, Second Dentition, p. 53, "In dissecting with especial care the matrices, one recognizes that they are composed of two membranes: the internal part of the mucous membrane which covers and enters into the composition of the gum, and extends to its termination at that line which separates the crown from the root, and which is called the neck of the tooth. * * * This exhales a mucous fluid, in the midst of which is placed merely the crown of the tooth. * * * The external wall of the matrix originates from the fibro-cartilaginous body which covers the alveolar border, and which enters into the composition of the gum; it descends, *enveloping the other*, and having with it a somewhat intimate union, as far as the neck of the tooth. Throughout this extent it is spongy, and has a rosy appearance, because one sees through it the small red vessels of the inner layer. Having reached this point, it leaves the latter, to spread itself upon the root as fast as this portion is formed; but all the time it envelops the base of the ganglion not yet ossified, and adheres to the dental nerve and artery which traverse it." Nasmyth's account is exactly similar: he says, Researches, &c. p. 110, where he is speaking of the tooth in the sacular stage, before its eruption, "The periosteum of the alveolus is the next of the structures in relation with the teeth, that calls for our examination. This membrane along the margin of the alveolus is closely connected with the mucous membrane of the mouth, and in this situation passes into the cavity of the alveolus and lines its surface. It is closely adherent to the osseous wall of the alveolus, and is furnished with blood vessels which pass into the substance of the bone. Internally it is connected with the external surface of the dental capsule, by means of loose areolo-fibrous tissue. As growth proceeds, and the dental capsule becomes thin, it unites with the periosteum, and the two membranes are inseparably blended together, forming only a single layer." And he immediately adds, "The coalescence of these membranes, and the thinning of their structure, which at the same time ensues, is associated with the production of the cortical substance of the tooth." Now we have only to remember that it is from the inner of these two membranes that the enamel-organ is formed, and that the lower portion of the outer membrane secretes the cement-pulp, and we find no difficulty in believing that the upward continuation of the same membrane, lying without the layer which gives origin to the enamel, may be charged with a similar function, and originate in its normal state the calcified membrane of Nasmyth, and in disease the monstrosity to which I have referred.

The radical portion of the cement is always more or less supplied with the characteristic lacunæ of bone, and when its quantity is large, it frequently presents true Haversian canals; so that its identity with osseous tissue is beyond cavil.

The purpose of this tissue varies with its situation. On the crown its function seems to be merely mechanical, for it resists sudden change of temperature far more effectually than the enamel which it covers, and thus protects it, in some measure, from fissure. But on the fang a much more essential duty is assigned it. The dentine, although highly endowed with capability of sensation, is yet a tissue of low vitality, and would be offensive to the more delicate structures which compose the jaw: hence a layer of intermediate qualities has been interposed; connected with the dentine on the one hand, by anastomosis with its tubuli, as well as by actual mechanical junction, and with the periosteum, on the other, by the nutritive dependence which it has upon that membrane. This office of the bony layer is beautifully exemplified in the condition of old roots, which have long been sources of irritation, as useless and dead members always are. They are found to be coated, very frequently, with an unusual quantity of cement; as if nature had attempted to cover the irritating body with an inoffensive investment, analogous to the capsule which forms around balls and other foreign bodies, which do not cause sufficient irritation to provoke a speedy expulsion.

It only remains to consider the tooth-pulp. As the dentine has been seen to be bone, modified to meet the requirements of the organ, so the pulp, from which primary and secondary dentine are formed, is cartilage, likewise modified to suit its function. And very different is this function from that of any other permanent cartilage; for the other cartilaginous masses have only mechanical offices, and their chief functional characteristic is elasticity, with a certain degree of softness. But no such property is needed in the dental pulp, and so totally different are its functions at maturity, that its character as cartilage seems chiefly intended to meet its histogenetic office, not only in the formation of primary dentine, but in many cases when subsequent conditions of the tooth call for additional deposits of tooth-bone, in some of its varieties.

The identity of tooth-pulp with cartilage is shown, not only by its conversion into dentine, but by its physical characters, and its microscopic appearances. We might expect that so highly vital an organ, so well protected from external violence, would be, as its name implies, soft and delicate; but the healthy pulp is very firm and tough under the dissecting needles, and cuts with no small degree of that peculiar feel which ordinary cartilage gives on section. Again, a thin section from the centre of the healthy pulp, shows, under the microscope, a structure very like that of cartilage; namely, a homogeneous matrix containing nucleated cells, scattered without apparent arrangement through its substance. True, the cells in this case are much smaller, and do not present the subdivisions—commonly fourfold—so characteristic of the common cartilage cell; but these are not essential differences, and the first impression made by a view of the pulp is "cartilage," while no other tissue at all approaches it in appearance.

But, though the connection cannot be doubted, there is a startling discrepancy in this cartilage, as compared with all others; for the smallest shred shows numerous bands of nerve-fibrils, and frequent bloodvessels, and as we approach the surface, these break up and ramify through the organ, endowing it with that intense vitality which is so remarkable in disease. These nervous bands are so numerous and broad, that they look, at first glance, like bands of white fibrous tissue, but the absence of the peculiar wavy appearance, and especially the unmistakeable characteristics of nerve-fibrils, which appear whenever a fibril is detached from its fellows, sufficiently identify this element; and neither variety of fibrous tissue occurs in the tooth pulp. If a fresh pulp, carefully removed from the dentine, be macerated for a day or two, the needles easily strip up from its surface a delicate membrane, and this, under the microscope, presents the characteristic cells, rather less thickly strewn, interlaced by the ultimate expansion of nerve-fibrils, with here and there a capillary vessel. It seems, indeed, less a distinct membrane, than a more delicate layer of the same structure as the pulp, containing the nerve-fibrils in a network rather than in bands. From this superficial layer proceed the tubes or fibrils of the dentine, which convey a certain nervous influence, either through actual nervous prolongations, or in a manner similar to that which obtains in the lowest orders of animals, whose structure is apparently homogeneous. Likewise the plasma of the blood is transmitted in such small quantities as are required by the extremely slight nutritive processes of the dentine.

Thus, then, we have glanced, in a cursory way, at some of the points which belong to the study of that admirable organ, the tooth—so perfect in its offices, so unobtrudingly useful, so terribly potent to punish neglect. We have tried to catch some hint of the primal conception—the *idea* of the Platonists—which guided its construction; we have seen how nature chooses rather to bend to new purposes the materials she already has at hand, than to create anew for every emergency. And yet we have gathered only a few simples from the vast field of research which these organs, so meanly estimated by the vulgar, offer to the patient student, who does not mistake humility for insignificance, nor need the sanction of popularity to enjoy the delightful retreats of nature's secret truths.

